# Spatial and temporal patterns in the population genomics of the European cockchafer *Melolontha melolontha* in the Alpine region

**DOI:** 10.1111/eva.13588

**Published:** 2023-09-01

**Authors:** Chiara Pedrazzini, Hermann Strasser, Niklaus Zemp, Rolf Holderegger, Franco Widmer, Jürg Enkerli

**Affiliations:** ^1^ Molecular Ecology, Agroscope Zürich Switzerland; ^2^ Institute of Environmental Systems Science ETH Zürich Switzerland; ^3^ Institute of Microbiology Leopold‐Franzens University Innsbruck Innsbruck Austria; ^4^ Genetic Diversity Centre (GDC) ETH Zürich Switzerland; ^5^ Swiss Federal Research Institute WSL Birmensdorf Switzerland

**Keywords:** ddRAD, European cockchafer, isolation by distance, mixed model, population genomic structure, temporal isolation

## Abstract

The European cockchafer *Melolontha melolontha* is an agricultural pest in many European countries. Populations have a synchronized 3 or 4 years life cycle, leading to temporally isolated populations. Despite the economic importance and availability of comprehensive historical as well as current records on cockchafer occurrence, population genomic analyses of *M. melolontha* are missing. For example, the effects of geographic separation caused by the mountainous terrain of the Alps and of temporal isolation on the genomic structure of *M. melolontha* still remain unknown. To address this gap, we genotyped 475 *M. melolontha* adults collected during 3 years from 35 sites in a central Alpine region. Subsequent population structure analyses discriminated two main genetic clusters, i.e., the South Tyrol cluster including collections located southeast of the Alpine mountain range, and a northwestern alpine cluster with all the other collections, reflecting distinct evolutionary history and geographic barriers. The “passo di Resia” linking South and North Tyrol represented a regional contact zone of the two genetic clusters, highlighting genomic differentiation between the collections from the northern and southern regions. Although the collections from northwestern Italy were assigned to the northwestern alpine genetic cluster, they displayed evidence of admixture with the South Tyrolean genetic cluster, suggesting shared ancestry. A linear mixed model confirmed that both geographic distance and, to a lower extent, also temporal isolation had a significant effect on the genetic distance among *M. melolontha* populations. These effects may be attributed to limited dispersal capacity and reproductive isolation resulting from synchronized and non‐synchronized swarming flights, respectively. This study contributes to the understanding of the phylogeography of an organism that is recognized as an agricultural problem and provides significant information on the population genomics of insect species with prolonged temporally shifted and locally synchronized life cycles.

## 1 INTRODUCTION

**FIGURE 1:**
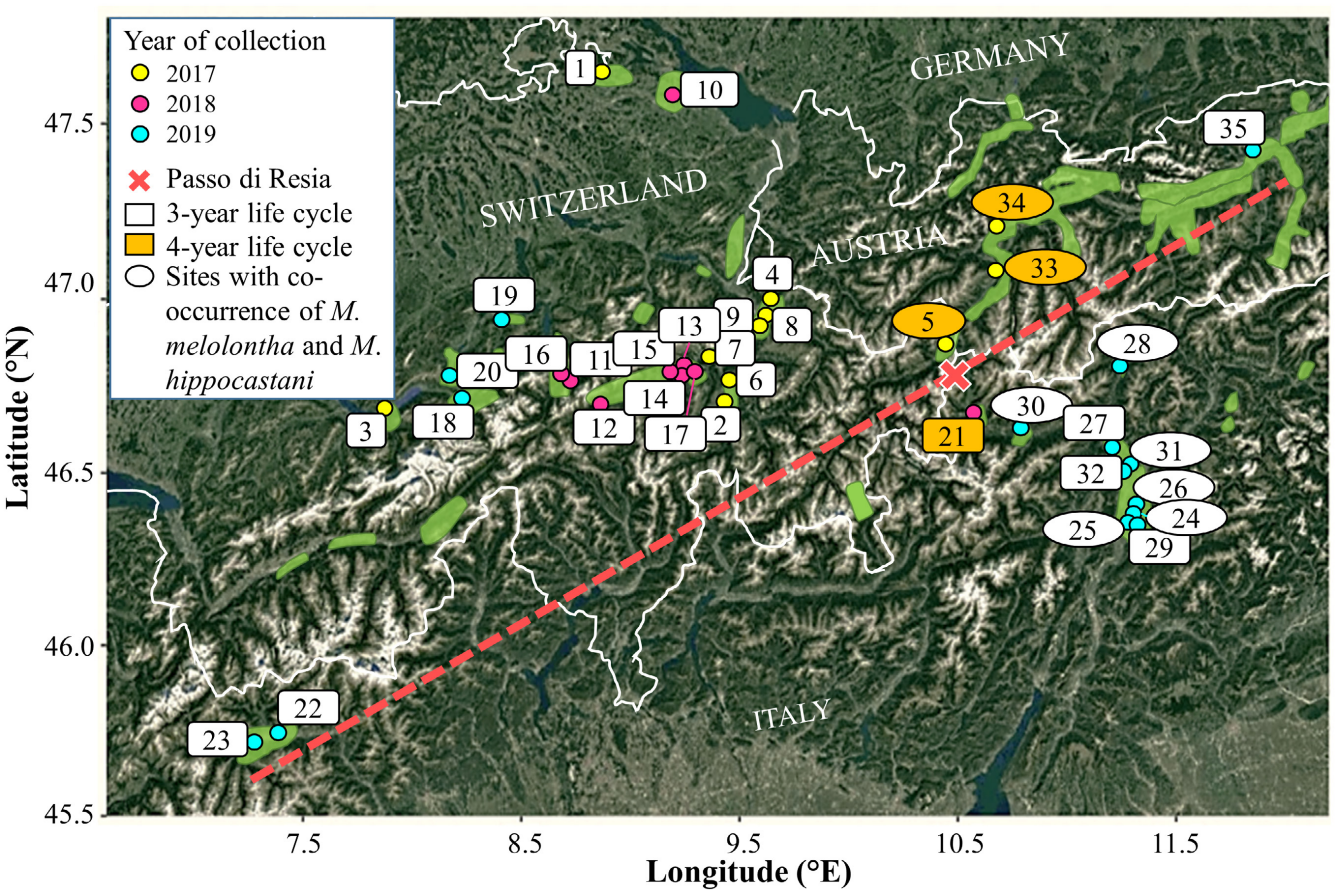
Map of collections of *Melolontha melolontha*, indicated by corresponding numbers given in Table S1. The white lines indicate the borders of Switzerland, Italy and Austria. Colours of the dots represent the year of collection of adults and the white and orange labels represent collections with a life cycle of 3 and 4 years, respectively. Numbers in ovals represent collections that were tested with the SNP‐based tool to exclude the presence of *M. hippocastani*. The dashed red line indicated the dividing line between the two main genetic clusters identified by STRUCTURE analyses (Figure 2b), and the red cross shows the location of the “passo di Resia” between North and South Tyrol (Figure 3). Areas of occurrence of *M. melolontha* are given in green.

The European cockchafer *Melolontha melolontha* L. (Coleoptera: Scarabaeidae) is a widespread insect throughout Europe, causing damage to agriculture and horticulture (Büchi et al., 1986; Tereba & Niemczyk, 2017; Wagenhoff et al., 2014). *M. melolontha* is considered native to Europe, where it represents the most prevalent *Melolontha* species in arable land (Freudiger, 1949; Kessler et al., 2004; Niemczyk et al., 2017; Poženel, 2005). According to the database of the European and Mediterranean Plant Protection Organisation (EPPO), *M. melolontha* is present in several European countries, with a high occurrence in central Europe (Figure 1; EPPO (2023) EPPO Global Database (available online). https://gd.eppo.int; Büchi et al., 1986; Poženel, 2005; Wagenhoff et al., 2014). Historical reports on infested areas and damage caused by the insect date back several centuries, e.g., in Switzerland first reports are available from 1478 (Freudiger, 1949). Most damage is caused by the soil‐dwelling polyphagous larvae of *M. melolontha*, also referred to as “white grubs” (Poženel, 2005; Sukovata et al., 2015; Woreta, 2015). While adults feed on the leaves or blossoms of trees and bushes, e.g., *Quercus robur* or *Acer pseudoplatanus*, larvae feed on roots of different tree and grassland species such as *Malus domestica* or *Taraxacum officinale*, but also on roots of arable crops, e.g., potato (Büchi et al., 1986; Laengle et al., 2005; Sukovata et al., 2015). The larval stage before pupation is the most voracious and causes the most serious damage; the damage threshold in grasslands is 10–30 larvae per m^2^, and in orchards or crops as low as 1–5 larvae per m^2^ (Keller et al., 1997).

*M. melolontha* larvae develop in soil by passing three larval instars, i.e., L1, L2 and L3 (Wagenhoff et al., 2014). After pupation, adults emerge in April to May, and swarm towards trees and along forest borders for a maximum distance of 2–3 km, feeding on tree leaves, which, in severe cases, can result in complete defoliation (Büchi et al., 1986). After mating, females return to the fields from which they have emerged for oviposition (Wagenhoff et al., 2014). *M. melolontha* generally completes its life cycle within 3 years, but in some alpine areas where average temperatures are lower the cycle can last for 4 years (Faber, 1951; Wagenhoff et al., 2014). Infested areas are typically inhabited by a population of *M. melolontha*, which is temporally synchronized, i.e., at the same development stage. In different areas and populations, adults can therefore emerge in different years, leading to the presence of yearly shifted and isolated populations. However, the simultaneous occurrence of different developmental stages in the same area has also been observed at some sites, and this can lead to temporally shifted populations in the same area or region (Faber, 1951; Enkerli and Strasser, personal observation).

Comprehensive monitoring for decades has provided extensive data on the presence of *M. melolontha* in countries such as Switzerland, Austria, Italy, Germany, Slovenia, Poland and France (Dolci et al., 2006; Keller et al., 1997; Laengle et al., 2005; Poženel, 2005; Richter, 1958; Robert et al., 1986; Sukovata et al., 2015). In regions heavily infested with *M. melolontha*, its abundance has been monitored for several decades and in some regions, a change in spatial occurrence of populations has been observed over the past 100 years (Zweigelt, 1928). In Switzerland, for example, large infested areas of about 15,000 km^2^ have been documented in the 1940s and 1950s at lower elevations of the Swiss Plateau, on the northern flank of the Alps and in southern Switzerland. During subsequent decades, infestations have shifted to central Switzerland, particularly to valleys and plains at altitudes between 300 and 1000 meters above sea level (Figure 1; Büchi et al., 1986). Which factors influence *M. melolontha* occurrence is uncertain, however, human activities like land exploitation and population control efforts as well as increasing temperature, e.g., in Switzerland temperatures have increased by 2°C since 1864, may have contributed to this shift (Federal Office of Meteorology and Climatology, MeteoSwiss (available online). https://www.meteoswiss.admin.ch; Büchi et al., 1986). In regions affected by *M. melolontha*, various approaches to control infestations have been proposed in the past, ranging from collecting individuals by the civil population in the 19th century to chemical control with Hexachlorocyclohexane (HCH) or Dichlorodiphenyltrichloroethane (DDT) from the 1940s to the 1970s (Straumann, 2005; Woreta, 2015). Due to environmental concerns, chemicals were banned for *M. melolontha* control, which promoted the development and commercialization of biological control approaches (Straumann, 2005; Woreta, 2015). The use of the entomopathogenic fungus *Beauveria brongniartii* has become the favoured and most efficient approach to control *M. melolontha* since the 1990s (Dolci et al., 2006; Keller et al., 1997; Laengle et al., 2005).

Despite extensive monitoring work in infested areas, knowledge on the genetic diversity of *M. melolontha* is limited and in‐depth analyses of its phylogeography and population structure are missing. Microsatellite markers, developed by Enkerli et al. (2008), have been used to study the genetic variation of *M. melolontha* and of the morphologically similar species *M. hippocastani*, which is also native and abundant throughout Europe and mainly inhabits forests (Büchi et al., 1986; Tereba & Niemczyk, 2017). *Melolontha* spp. individuals were collected from three forest areas in central and southeastern Poland and genetic analysis based on eight polymorphic markers revealed a higher observed heterozygosity in *M. melolontha* than in *M. hippocastani*, but the comparison was not reliable as fewer alleles were found for the latter species (Tereba & Niemczyk, 2017). The mitochondrial cytochrome c oxidase (CO1) subunit 1 gene has been used as a molecular marker for phylogenetic studies on *Melolontha* spp. and for developing a SNP‐based tool to discriminate *M. melolontha* from *M. hippocastani* (Giannoulis et al., 2011; Pedrazzini et al., 2021). Furthermore, microsatellite markers and CO1 sequences have been used to investigate the genetic variation in *M. melolontha* and *M. hippocastani* in five pine forests from central and eastern Poland, which revealed no population differentiation for neither species (Masternak et al., 2022). Despite the previously mentioned studies, to our knowledge, there are currently no genomic studies based on high‐resolution molecular markers on *Melolontha* spp. available covering a wider geographic area and including individuals collected during temporally separate swarming flights. *M. melolontha* offers the great opportunity to study the genomic structure of populations emerging in different years, an aspect that is still insufficiently investigated in insect species. Population genomics could provide valuable information on the dynamics within populations, allowing the identification of key factors influencing *M. melolontha* distribution.

The aim of this study was to address the following three topics: (1) analysing the population structures of *M. melolontha* collected at infested sites in the Alpine region of Switzerland, Austria and Italy, in order to (2) determine the effect of spatial separation and (3) temporal isolation on the genetic distance among collections of the European cockchafer, *M. melolontha*. Analyses were based on genome‐wide single nucleotide polymorphism (SNP) loci obtained from double‐digest Restriction site Associated DNA sequencing (ddRADseq).

## 2 MATERIALS AND METHODS

### 2.1 Collection of individuals and DNA extraction

From 2017 to 2019, collections of *M. melolontha* beetles were established from 35 infested sites, including 20 sites in Switzerland, 12 sites in Italy and three sites in Austria (Figure 1; Table S1). The map of *M. melolontha* occurrence was constructed with the R package GGMAP 3.0.0 in R version 4.2.2 (Kahle & Wickham, 2013; Team, 2020). Following collection and transport of alive *M. melolontha* adults to the laboratory, beetles were frozen and stored at −80°C. The legs of each individual were homogenized at 30 Hz for 2 × 30 s at RT using a TissueLyser II (QIAGEN) and two 3 mm NucleoSpin® steel beads (Macherey & Nagel). DNA was extracted using the LGC sbeadex Plant Kit (LGC) automated with the KingFisher Sample Purification System (Thermo Fisher Scientific). DNA quality was assessed visually after electrophoresis in 1%‐agarose gels and quantified with PicoGreen® fluorescent nucleic acid stain (Invitrogen).

### 2.2 Exclusion of *M. hippocastani* from insect collections

Co‐occurrence of the species *M. melolontha* and *M. hippocastani* was documented at nine sampling sites (i.e., Strada, Branzoll, Schoenwies, Passeier‐Sandwirt, Prutz, Siebeneich, Laimburg, Schlanders and Kaltern‐OG Roen; Figure 1). A previously developed SNP‐based tool detecting species‐specific SNPs in a fragment of the mitochondrial CO1 gene was applied for species identification with subsequent exclusion of *M. hippocastani* individuals from the collections (Pedrazzini et al., 2021). Missing samples were complemented with DNA extracted from additional individuals to obtain 15 *M. melolontha* individuals per site used for library production.

### 2.3 ddRADseq library preparation and sequencing

DNA extracts from 525 samples were randomized on 96‐well plates using a PIPETMAX® (Gilson). The ddRADseq libraries were prepared with 240 ng DNA per sample according to Westergaard et al. (2019). Genomic DNA was double‐digested with EcoRI‐HF® and TaqI‐v2 restriction enzymes (New England Biolabs) and ligated with T4 DNA Ligase (New England Biolabs) to biotinylated Illumina barcoded adapters. Each library comprised multiplexed DNA of 46‐barcoded individuals as well as a positive and a negative control. A 500 bp size‐selection was performed for each library using 0.64× Agencourt AMPure XP beads (Beckman Coulter), to obtain a collection of fragments of 400–700 bp. Subsequently, libraries were washed and purified using M‐270 Dynabeads® magnetic beads (Thermo Fisher Scientific) selecting for P2‐biotin labelled adapters. To enrich libraries with Illumina indexes, a PCR amplification was performed using the Phusion® High‐Fidelity PCR Master Mix with HF Buffer (New England Biolabs; Table S2). Cycling conditions consisted of an initial denaturation of 120 s at 95°C, followed by 10 cycles of 20 s at 98°C, 20 s at 65°C and 30 s at 72°C. Libraries were quantified with a Qubit 2.0 fluorometer (HS dsDNA kit, Thermo Fisher Scientific) and fragment‐size assessed on an Agilent 2200 Tape Station. Libraries were sequenced using the NovaSeq 6000 platform with 150 bp paired‐end reads, yielding 1–8 Mio reads per sample with a mean tenfold coverage (Novogene). We deposited raw data at ENA under accession number PRJEB60431.

### 2.4 Sequence quality control, de‐novo assembly, variant calling and SNP filtering

Raw sequences were demultiplexed applying the *process_radtags* component of STACKS 2.55 using default settings (Catchen et al., 2013). High‐quality genome‐wide SNP markers were detected following the DDOCENT pipeline (Puritz et al., 2014). A reference catalogue of RAD loci was assembled previously from 24 randomly chosen individuals who were sequenced on the Illumina MiSeq platform with 300 bp paired‐end reads (Genetic Diversity Centre, Zürich, Switzerland). Long MiSeq reads were merged for the assembly. The best reference catalogue was constructed by testing different combinations of parameters to maximize the remapping rate of the reads, i.e., by varying coverage of unique sequences within individuals, number of shared unique sequences among individuals and similarity parameter for clustering. Optimal re‐mapping rate was achieved according to the following criteria: unique sequences had to be present at least three times within individuals and at least in two individuals. Sequences were clustered employing a first similarity threshold of 93%, and a second similarity threshold of 99%, resulting in a reference catalogue including 98,665 fragments. After construction of the reference catalogue, demultiplexed NovaSeq reads were mapped to it using BWA 0.7.17 (mean coverage 10×; Li, 2013). After that, a SNP panel was made by randomly selecting two individuals per collection. SNPs were detected using FREEBAYES 1.3.1 (Garrison & Marth, 2012) and filtered using VCFTOOLS 0.1.16 (Danecek et al., 2011) and VCFLIB 1.0.1 (Garrison, 2012). Only SNP loci that satisfied the following criteria were considered: (1) a minimum quality score of 20, (2) a minor allele count of three, (3) a minimum mean depth of three, (4) a mean depth of 10, (5) a minor allele frequency of 1% and (6) successfully genotyped in 50% of individuals. Individuals with more than 50% missing sites were excluded from analysis. Loci with more than 20% missing data per population, complex SNPs and indels were removed. SNPs were haplotyped using RAD HAPLOTYPER (Willis et al., 2017) to remove potential wrongly assembled loci, and 43,164 high‐quality SNPs were obtained. After that, SNPs of the panel were re‐genotyped in the entire dataset (475 individuals) applying the same filters as previously mentioned, but with 5% minor allele frequency and only one biallelic SNP per RAD locus (1 kilobase apart) was retained to reduce issues of linkage disequilibrium. A total of 8358 SNP sites were identified among the 475 *M. melolontha* adults. The software PGD SPIDER 2.1.1.5 (Lischer & Excoffier, 2012) was used for the conversion of the final vcf file to other formats.

### 2.5 Genetic diversity and population genomic structure

The method of Schmidt et al. (2021) was applied to comprehensively assess heterozygosity, i.e., observed (*Ho*) and expected heterozygosity (*He*), which considers both monomorphic and polymorphic nucleotides for more robust estimation. For each collection, nine individuals were randomly selected to account for differences in sample size between collections. Estimates were extracted from the STACKS population summary statistics output run including individuals with 0% missing data. The R package VCFR 1.13.0 was used to import vcf files into R (Knaus & Grünwald, 2017). Pairwise F_ST_ values based on allele frequencies were calculated among the 35 collections with the R package STAMPP 1.6.3 (Pembleton et al., 2013). Overall genomic structure in the dataset and individual admixture proportions were performed with the Bayesian clustering approach implemented in STRUCTURE 2.3.4 (Porras‐Hurtado et al., 2013; Pritchard et al., 2000). Analyses were run based on the admixture model for K values ranging from one to 10 with 10 replicate runs, and burn‐in iterations of 5000 generations followed by 10,000 Markov Chain Monte Carlo (MCMC) repetitions. For visualization of the estimated population structure, runs were clustered and averaged using CLUMPAK (Kopelman et al., 2015). The inference of the true K‐value in STRUCTURE is often difficult, as there may be different optimal K‐values corresponding to different hierarchical levels. Hence, a range of K‐values were analysed and visually inspected (Funk et al., 2020; Lawson et al., 2018). Q‐matrices were imported into R and maps with assignment membership were constructed with the R packages RWORLDMAP 1.3–6 and MARMAP 1.0.6 (Pante & Simon‐Bouhet, 2013; South, 2011). As a complementary approach to identify and visualize genetic clusters of the data, a Discriminant Analyses of Principal Components (DAPC) was run with ADE4 1.7–18 (Dray et al., 2007) and ADEGENET 2.1.5 (Jombart, 2008) without prior group information, with the function *find.cluster*. DAPC has no population genetic assumptions (e.g., linkage equilibrium or Hardy–Weinberg equilibrium), employs sequential K‐means, and relies on the Bayesian information criterion (BIC) to infer genetic clusters (Jombart et al., 2010). In addition and to visualize genomic differentiation between collections from different sampling sites, a DAPC with a priori population designation (i.e., sampling sites) was performed with the R package ADEGENET 2.1.5 (Jombart, 2008) and a UPGMA dendrogram based on Nei's genetic distance was built with the R package POPPR 2.9.3 (Kamvar et al., 2014) with 1000 bootstrap replicates.

### 2.6 Isolation by distance and linear mixed model

Pairwise Nei's genetic distance among collections of *M. melolontha* was calculated using the R package ADEGENET (Jombart, 2008), and the Euclidean geographic distance matrix was obtained with the R package REAT 3.0.3 (Wieland, 2020). To test for the presence of Isolation By Distance (IBD), a Mantel test was performed using the R package VEGAN 2.6‐2 (Oksanen et al., 2013) with 10,000 permutations between pairwise genetic distance and pairwise geographic distance matrices and visualized with the R package GGPLOT2 3.3.5 (Wickham et al., 2016). The EEMS (Estimating Effective Migration Surfaces) algorithm was employed to examine spatial patterns of gene flow over a long evolutionary time scale of *M. melolontha*, highlighting regions with enhanced or restricted gene flow (Petkova et al., 2016). A dissimilarity matrix was calculated following Petkova et al. (2016), and the *runners_snps* function in EEMS was used to estimate effective migration rates and generate a migration surface. The algorithm was run for 2,000,000 MCMC iterations, with a burn‐in of 1,000,000 iterations and a thinning interval of 9999.

The effect of geographic and temporal distances on genetic distance was further investigated with a linear mixed effect model (LMM) approach that is increasingly used in ecology (Milanesi et al., 2017; Van Strien et al., 2012) implemented in the R package MCMCGLMM 2.34 (Hadfield et al., 2010). In the LMM, the pairwise Nei's genetic distance matrix previously calculated for the IBD test, measuring genomic differentiation between collection pairs, was used as response variable. The fixed effect variables in the model were the predictor matrices: geographic distance (Euclidean distance) and temporal isolation. Geographic distance quantified spatial separation, while temporal isolation distinguished between temporally synchronized (1) and shifted (0) collections. These variables were included to investigate the effect of spatial and temporal factors on genomic differentiation. Due to non‐independence of genetic distances, i.e., each collection is used for calculation of many pairwise genetic distances, a random effect was introduced to appropriately account for multiple membership of the collections compared. Four models were run to investigate the effect of geographic separation and temporal isolation on the genetic distance among collections: a null model, i.e., including only the random effect, a model adding geographic distance as fixed effect term, a third model adding temporal isolation as fixed effect term, and a fourth model adding both geographic distance and temporal isolation as fixed effects. The models were run with standard priors and a burn‐in of 500,000, 2,000,000 MCMC iterations, and a thinning interval of 750. Model comparison was performed using the Deviance Information Criterion (DIC) and the most suitable model, i.e., the model with the lowest DIC value, was checked for convergence, and low autocorrelation with the *autocorr.diag* function of the R package CODA 0.19–4 (Plummer et al., 2006). In addition, a boxplot representing pairwise genetic distance in synchronized and non‐synchronized collections was constructed with GGPLOT2 3.3.5 (Wickham et al., 2016), and significant differences were tested with a two‐tailed *t* test using the R package STATS 4.2.2 (Team, 2020).

## 3 RESULTS

### 3.1 Data set used in this study

Following mapping to the reference catalogue and SNP filtering, 50 *M. melolontha* individuals that did not satisfy the quality criteria were excluded from further analyses, resulting in a final dataset of 475 individuals from 35 collections established in three different years, with 9–15 individuals per sampling site (Figure 1; Table S1).

### 3.2 Analysis of population structure

Observed heterozygosity (*Ho*, 0.8 × 10^−4^–1.7 × 10^−4^) was similar to expected heterozygosity (*He*, 0.7 × 10^−4^‐1.6 × 10^−4^) at all 35 sites. Mean values of *Ho* and *He* were overall small and similar across collections, but mean *Ho* values were slightly lower in the 10 South Tyrolean collections (i.e., 0.8 × 10^−4^). Highest levels of heterozygosity were detected in the two collections from the Aosta Valley, i.e., *Ho*, 1.6 × 10^−4^ and 1.7 × 10^−4^ (sites 22 and 23; Table S3, Figure S1). Values of observed and expected heterozygosity showed no discernible temporal pattern, indicating relatively stable levels of genetic diversity across sampling years. Pairwise F_ST_ values revealed low levels of genomic differentiation among the 35 collections of *M. melolontha* (i.e., 0–0.067), suggesting a high degree of genetic similarity.

**FIGURE 2:**
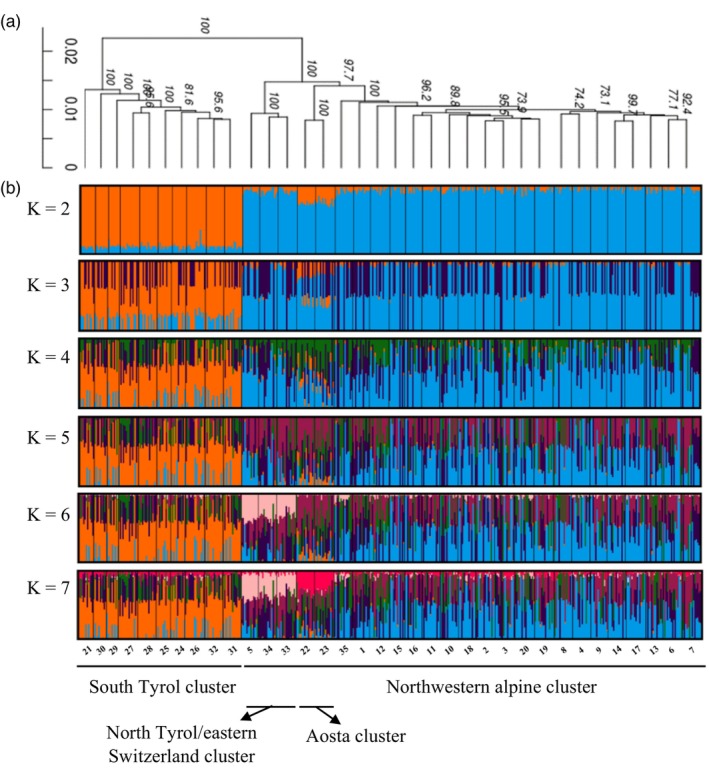
Dendrogram based on Nei's genetic distance including 35 collections of *Melolontha melolontha* (high‐bootstrap values >70% are labelled at major nodes, 8358 SNPs, *N* = 475); (a) and admixture plots (10 runs per K) obtained from STRUCTURE and CLUMPAK analyses from K = 2 to K = 7 (b). Each vertical line represents an individual, and the colour is proportional to the membership coefficient (*Y*‐axis) to the K clusters. Identified genetic clusters are indicated at the bottom of the figure.

A dendrogram constructed by including all 35 collections supported the presence of four genetic clusters (Figure 2a). High bootstrap values supported the separation of the 10 South Tyrolean collections from the remaining collections. Prutz (33: Austria), Schoenwies (34: Austria) and Strada (5: Switzerland) were grouped together in a separate clade, as were Aosta‐1 (22: Italy) and Aosta‐2 (23: Italy). Except for the collection Muenster (35: Austria), which clustered with 19 Swiss collections, the dendrogram reflected the geographic origin of the collections well (Figure 2a).

**FIGURE 3:**
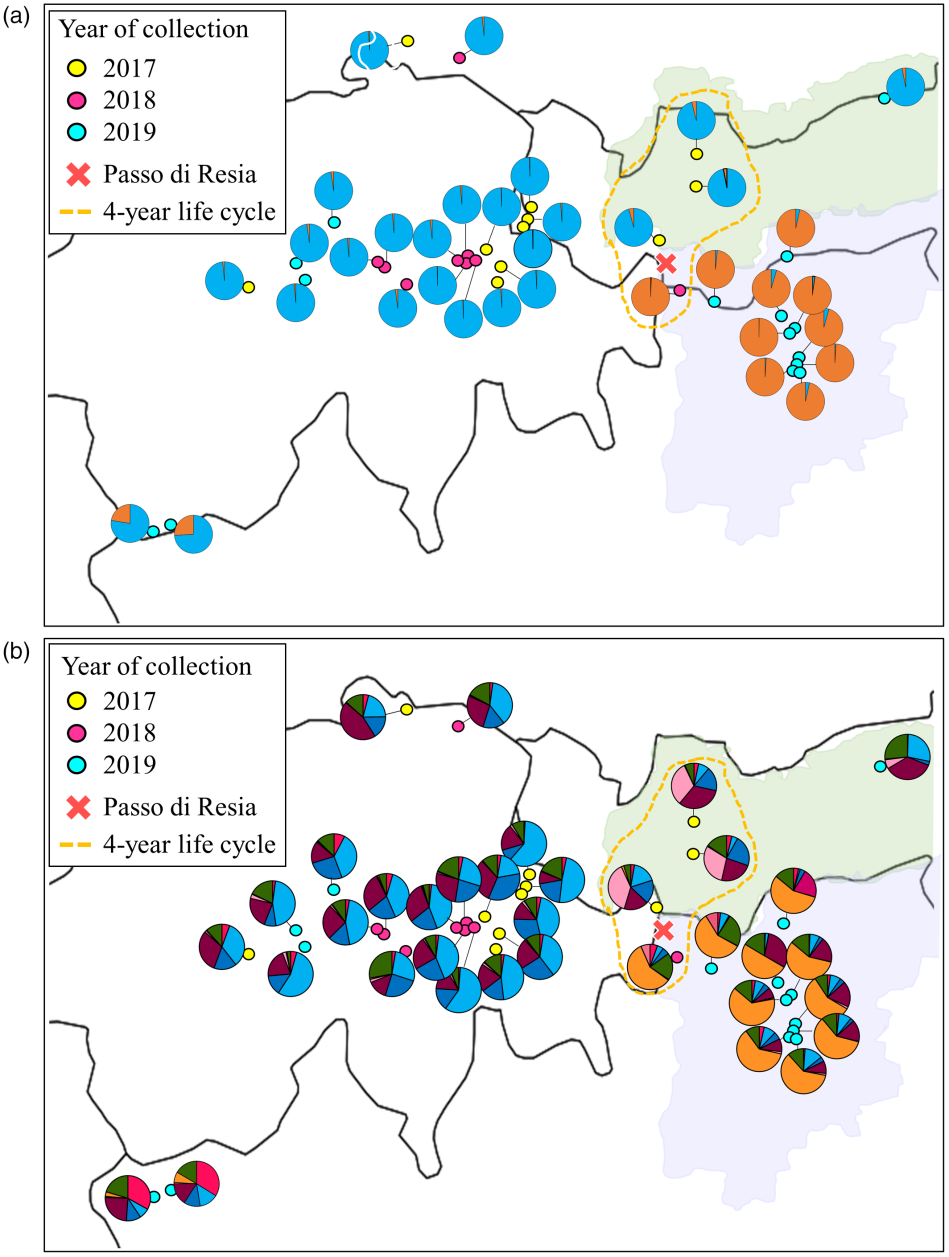
Map of collection of *Melolontha melolontha*. Collection sites of individuals and their affiliation to different genetic clusters at K = 2 (a) and K = 7 (b). Coloured points indicate year of sampling sites, and pie charts represent relative membership coefficients to genetic clusters inferred by STRUCTURE and CLUMPAK analyses. The two regions of South and North Tyrol are indicated by violet and green shades, respectively, and the “passo di Resia” between the two areas is indicated with a red cross.

Analyses of genetic clusters using STRUCTURE revealed relevant genomic structure for K = 2, where 23 collections, i.e., 1–20; 33–35 (Table S1) were assigned to a “northwestern alpine” cluster with an average membership coefficient of 0.985, and 10 collections from South Tyrol in northern Italy (i.e., 21; 24–32; Table S1), were allocated to a “South Tyrol” cluster, with an average membership coefficient of 0.980 (Figures 2b, 3a). The collection Glurns (i.e., 21) with 4‐year life cycle clustered with the other collections from South Tyrol with a 3‐year life cycle. Two collections from northwest Italy (22: Aosta‐1, 23: Aosta‐2) had an averaged membership coefficient of 0.758 for the northwestern alpine cluster and 0.241 for South Tyrol cluster, hence partly exhibiting ancestry with South Tyrolean collections (Figures 2b, 3a). A high proportion of admixed individuals was detected as the number of clusters increased (i.e., K = 3–5), but the separation between the South Tyrol collections and all other collections was still evident (Figure 2b). The presence of the four main genetic clusters observed in the dendrogram was also detected by the STRUCTURE analysis (Figure 2). At K = 6 and K = 7, collections from South Tyrol were still well separated from all other collections, and the collections from Prutz (33: Austria), Schoenwies (34: Austria) and Strada (5: Switzerland) were partly assigned to a different cluster (Figures 2b, 3b). Furthermore, at K = 7 the collections from Aosta (22: Aosta‐1, 23: Aosta‐2) showed a high membership coefficient to a separate cluster (Figures 2b, 3b). From K = 7 to K = 10, no additional consistent clusters were identified (data not shown). The Bayesian Information Criterion (BIC) reached its lowest point at K = 3 (Figure S2a). At K = 2, DAPC analyses without prior group information identified the same genetic clustering as STRUCTURE at K = 2, with all individuals from South Tyrol assigned the South Tyrol cluster, and the remaining individuals assigned to the northwestern alpine cluster (Figure S2b). At K = 3, the clustering of South Tyrol samples was consistent, whereas the remaining individuals were assigned to either cluster 1 or cluster 3, and the DAPC scatterplot showed high genomic differentiation of cluster 2 from clusters 1 and 3 (Figure S2c).

Population genomic structure observed by the dendrogram, was also confirmed by the DAPC analyses performed with a priori group information (Figure S3).

### 3.3 Isolation by distance and linear mixed model

Geographic distance among collection sites ranged from 3 to 398 kilometres and pairwise Nei's genetic distance, calculated among the 35 collections, ranged from 0.016 to 0.053. The highest genetic distance was found between an Austrian collection (34: Schoenwies) and a South Tyrolean (29: Plattl) collection, while the lowest genetic distance was found between two Swiss collections (14: Ilanz, 17: Valendas). A Mantel test performed between Nei's genetic distance and geographic distance matrices revealed a significant positive correlation (*r*: 0.49, *p*: 0.001; Figure S4a). A significant positive correlation was also determined in a dataset excluding the 10 collections from South Tyrol (*r*: 0.62, *p*: 0.001; Figure S4b). Based on the EEMS analyses, high dispersal was observed among sites in northwest Italy (22: Aosta‐1, 23: Aosta‐2), central eastern Switzerland, as well as in South Tyrol, while the remaining sites showed low‐migration rates (Figure S5).

The Deviance Information Criterion (DIC) revealed that the model including both geographic distance and temporal isolation as explanatory variables was the most suitable; the DIC value of this model was lower than the values obtained for models including either none, only geographic distance or only temporal isolation as explanatory variables (Table S4). The model indicated that both geographic separation and temporal isolation weakly explained the pattern of genetic distance across the 35 collections. The estimated intercept, representing the baseline genetic distance when other variables are not considered, was 2.178 × 10^−2^. The model detected a positive significant (*p* < 0.005, estimated coefficient 9.083 × 10^−5^) association between geographic and genetic distance. Temporal isolation exhibited a negative significant (*p* < 0.005, estimated coefficient − 3.737 × 10^−3^) association with genetic distance. In accordance with the model, genetic distance was slightly, but significantly higher between collections separated by a larger distance, and significantly, though slightly lower among collections from sites where adults were swarming in the same year (Figure S6).

## 4 DISCUSSION

The present study was performed to investigate the population genomic structure of *M. melolontha* in an Alpine region, which is particularly affected by severe crop and grassland damage caused during infestations (Figure 1). It provided information on the intraspecific temporal and geographic genomic variation of this species using SNP markers that revealed population differentiation despite admixture and low‐pairwise F_ST_ values. Furthermore, it provided evidence that both geographic and temporal separation affect genetic distance among collections of *M*. *melolontha*.

Bayesian STRUCTURE, DAPC analyses and the dendrogram discriminated the 10 South Tyrolean collections of *M. melolontha* from the other 25 collections, a separation that persisted irrespective of the number of K considered (Figure 2; Figure S2). The separation of the two main genetic clusters coincided with topographic elements and, in particular, with the mountain ranges separating the South Tyrol region (i.e., Vinschgau, South Tyrolean Unterland and Burggrafenamt) from surrounding areas (Figure 1). Topographic barriers causing strong genomic differentiation between natural populations are known to exist in the Alpine region (Schönswetter et al., 2002; Thiel‐Egenter et al., 2009, 2011). Genomic differentiation between collections from the western and eastern parts of the central Alps has been reported in population structure studies of insect species, such as the copper butterfly *Lycaena hippothoe*, the mountain butterfly *Erebia alberganus*, and the mountain cadmietta *Drusus discolor*, indicating the presence of genetic barriers in the central part of the Alps (Louy et al., 2014; Pauls et al., 2006; Trense et al., 2022). Glaciation events that occurred in the Pleistocene caused changes in the geographic distribution of numerous species and may be the reason for genomic differentiation between eastern and western populations of alpine species (Hewitt, 2004; Schmitt, 2009; Thiel‐Egenter et al., 2011). Evidence from different studies shows that during glaciation, many species remained isolated in various glacial refugia, and expanded after glaciation from these refugia giving rise to different genetic lineages, which then faced dispersal barriers resulting in genetic breaks (Schönswetter et al., 2005; Thiel‐Egenter et al., 2011). In the present study, the collections from South Tyrol formed a distinct genetic cluster, indicating a genomic structure specific to that region. Conversely, the collections from the northern part of Tyrol displayed a genomic structure which was related to the collections from Switzerland. This observation highlights the presence of a barrier limiting genetic exchange between South Tyrol and northwestern Alps, contributing to the genomic differentiation observed in the study (Figure S5). The mountain ranges dividing South Tyrol region from northern Alps may have acted as physical barriers to dispersal of *M. melolontha* adults, as supported by the low‐migration rates detected between these regions (Figure 1; Figure S5). *M. melolontha* generally occurs on valley floors, where climatic conditions are mild and favourable (Büchi et al., 1986; Faber, 1951). Therefore, topographical features such as mountains, where temperatures are generally lower, may limit the movement and dispersal of *M. melolontha* adults and act as barriers to gene flow. The two detected genetic clusters (i.e., northwestern alpine and South Tyrol) might represent separate genetic lineages, which recolonized the studied alpine region from different refugia. The collections from northwest Italy (Aosta, 22 and 23), although belonging to the northwestern alpine cluster, showed admixture with South Tyrolean genotypes at K = 2, whereas at K = 7, they showed membership coefficient to a separate cluster (Figures 2b, 3). These results suggest reduced gene flow between the Aosta and South Tyrolean collections, which is also supported by the presence of a region of low‐migration rate in between, however, with potential progressing interbreeding between intermediate populations in northern Italy (Figure S5). More in depth analyses of individuals from the Lombardy and Piedmont regions, which connect Aosta and South Tyrol, would be required to provide a clearer understanding of the connectivity between these populations and shed light on the genomic structure of *M. melolontha* in this region. Three collections from North Tyrol or eastern Switzerland (33: Prutz, 34: Schoenwies and 5: Strada) showed shared ancestry at K = 7 (Figures 2b, 3b). The three sites are 40 kilometres apart, with a documented continuous presence of *M. melolontha* also in between the sampled sites. Monitoring over the last few decades has shown that individuals from North Tyrol have a synchronized life cycle of 4 years, while swarming flights are observed every year in the southeastern location Strada (5) in Switzerland, with some years having larger peaks (Steivan Martinelli, Strada; personal communication). Since *M. hippocastani*, which is morphologically similar and has a life cycle of 4 years, is also documented in this region (Figure 1), *M. melolontha* monitoring might have been hampered by inaccurate species identification in the past. According to the results of this study, however, the individuals from Strada are genetically similar to those from Prutz and Schoenwies, suggesting that they belong to the same population (Figures 2b, 3b). In contrast, the collection from Northeast Tyrol (35: Muenster) exhibited a distinct clustering pattern by grouping with the Swiss collections, suggesting reduced gene flow with Strada, Prutz and Schoenwies (Figure 2b).

The genomic separation between the South Tyrol and the three previously mentioned collections from North Tyrol and eastern Switzerland (33: Prutz, 34: Schoenwies and 5: Strada) was particularly interesting, because despite the geographic proximity of these collections, which is approximately 15 km, individuals were assigned to different STRUCTURE clusters (Figures 2b, 3b). The two regions are located at the ends of two valleys separated by an artificial lake, i.e., “Lago di Resia” (1498 meters above sea level), built in the 1950s by joining two natural lakes, and by the mountain pass “passo di Resia” of 1504 meters above sea level. Occurrence of *M. melolontha* between the two regions of South Tyrol and North Tyrol has never been reported, neither in recent decades nor one hundred years ago (Zweigelt, 1928), and there is no stable population of *M. melolontha* on the pass (Hermann Strasser, personal observation). Studies on the grasshopper *Chorthippus parallelus*, aimed at investigating the genomic differentiation of individuals in the same region of the “passo di Resia”, revealed levels of differentiation at the cytogenetic and genetic level between northern and southern collections along the pass. The authors hypothesized that this finding was the result of a postglacial expansion of *C. parallelus* from at least one Balkan refugium to the north of the Alps and from one refugium in southern Italy to the south of the Alps (Flanagan et al., 1999; Hagberg et al., 2022). Our data suggest that *M. melolontha* may show a similar phylogeographical pattern, with one genetic lineage represented by the South Tyrolean collections and a second genetic lineage represented by the North Tyrolean and all other collections (Figure 2). The altitude of the pass, combined with the low temperatures, may have acted as a physical barrier hindering the survival and development of *M. melolontha* during colder months, and limiting its presence in the region. There is only limited information available on the dispersal distance of *M. melolontha* adults, but it is assumed that they do not fly more than 2–3 km and remain within the proximity of the grasslands or fields where they emerged (Büchi et al., 1986). Therefore, it seems that a geographic distance of only about 15 km is sufficient to prevent or strongly reduce gene flow. In addition to the physical barrier of the “passo di Resia”, the temporal non‐synchronization between the collections north of the pass (i.e., 4 year life cycle, collected in 2017) and the geographically closest collection Glurns (21) in South Tyrol (i.e., 4 year life cycle, collected in 2018) might also have acted as an additional barrier, preventing interbreeding among individuals from north and south of the pass.

The linear mixed model revealed that both temporal and geographic separation have an effect on the genomic differentiation of *M. melolontha* species in the Alpine region studied. An increase in genetic distance with increasing geographic distance has been observed (isolation by distance, IBD; Figure S4a). Interestingly, the low‐pairwise F_ST_ genetic distance values suggested that substantial gene flow occurred among *M. melolontha* collections (i.e., 0–0.067), despite isolation by distance and the presence of regions of limited gene flow represented in Figure S5. A pattern of IBD was also observed among the collections of the northwestern alpine genetic cluster when the South Tyrolean cluster was excluded for analysis (Figure S4b). Studies on Coleoptera species with great dispersal capacity have shown a weak pattern of isolation by distance, such as in *Ips typographus* along a transect with a diameter of 300 km in Sweden (Ellerstrand et al., 2022), in *Ootheca mutabilis* along a transect of 300 kilometres in Uganda (Kanyesigye et al., 2022), or in *Cerotoma trifurcata* along a transect of 1545 km in the USA (Tiroesele et al., 2014). However, the results of the present study indicate, even though genetic distance values across collections were low, that geographic distance had an effect on the genomic structure of *M. melolontha*, along a transect of 398 km. Since *M. melolontha* has limited dispersal capacity (see above), interbreeding is more likely between individuals from the same area and less frequently occurs between geographically distant individuals, implying that gene flow tends to be greater between geographically adjacent collections. However, the isolation by distance model is simplistic and other factors, e.g., phylogeography, landscape elements, temporal isolation and anthropogenic activities like pesticide use or agricultural intensification can influence population differentiation within species (Milanesi et al., 2018; Ribolli et al., 2017; Thiel‐Egenter et al., 2011).

According to the results of this study, temporal separation also had an effect, although small, on the genetic distance among collections. Temporally synchronized collections (i.e., exhibiting a synchronized swarming flight regardless of the length of their life cycle) were characterized by slightly smaller genetic distances between them, suggesting that the length and timing of the life cycle contributes to population differentiation (Figure S6). Population differentiation based on different breeding and/or spawning time, regardless of spatial location, has been observed in several species, such as in the migratory fish *Salminus brasiliensis* (Ribolli et al., 2017) and the frog *Rana arvalis* (Richter‐Boix et al., 2013). Although still little studied, temporal population genomic structure has also been observed in insects with long life cycles or multiple emergence periods, such as the butterfly *Neophasia menapia* (Bell et al., 2017) and the moth *Thaumetopoea pityocampa* (Santos et al., 2011). In *M. melolontha*, the temporal synchronization of the life cycle allows reproduction between adults during swarming flights, whereas collections with a non‐synchronized life cycle would not reproduce due to incompatible developmental stages. Reproductive isolation would prevent gene flow and increase genomic differentiation between populations with a shifted life cycle. The difference in genetic distance between the synchronized and non‐synchronized collections was very low, indicating that considerable gene flow had occurred between the temporally shifted collections. This result suggests that currently non‐synchronized populations of *M. melolontha* may have been synchronized in the past but suffered a change in temporal dynamics, perhaps due to changes in temperature or area of occurrence, causing the current life cycles to be shifted in time. Low‐genetic distance between non‐synchronized collections might also result from the fact that currently non‐synchronized populations of *M. melolontha* interbreed at regular intervals over the years, such as collections with 3‐ and 4‐year life cycles synchronize every 12 years, allowing interbreeding and gene flow to occur. This is illustrated by the South Tyrol collections, where 10 collections belonged to the same genetic cluster, but nine collections at 217–808 m altitude had a temporally synchronized life cycle of 3 years, while one collection (21: Glurns), located at 1074 m altitude, had a life cycle of 4 years (Figure 1). The low‐genetic distance between the nine South Tyrolean and Glurns collections, which have a temporally shifted life cycle, may be explained by the fact that the life cycles of the 10 collections synchronize every 12 years, when gene flow may occur, provided that their dispersal abilities allow interaction of the beetles from these sites. However, the low‐genetic distance of the Glurns collection could derive from the Glurns collection originating from the nine valley floor collections. This hypothesis is supported by observations from various Alpine regions indicating that *M. melolontha* is colonising an increasing number of locations at higher altitudes in recent years (Hermann Strasser and C. Schweizer, Agroscope, personal communication). The Glurns collection area could thus have been colonized from sites with a 3 year life cycle at a lower altitude, and the movement of adults to higher altitudes with lower average temperatures might have decelerated the life cycle to 4 years. Faber (1951) already reported a change in the life cycle of a population of *M. melolontha* in Tyrol, from three to 4 years, due to the movement of individuals to higher altitudes. Insects are sensitive to environmental factors, and fluctuations in temperature directly affect their physiology and life cycle (González‐Tokman et al., 2020; Iannella et al., 2020; Illich & Zuna‐Kratky, 2022). However, while lower temperatures prolong the life cycle and delay egg maturation, higher temperatures accelerate the life cycle in insect species, resulting in faster development and earlier emergence, as observed for *M. melolontha*, where more frequent egg‐laying and shorter developmental stages have been observed (Büchi et al., 1986; Stefanescu et al., 2003). This is a relevant aspect to consider, especially since the worldwide trend of gradually increasing temperatures is accentuated in the Alpine region (Illich & Zuna‐Kratky, 2022). Under a global warming scenario, the beetle is expected to move to higher altitudes and, as temperatures are expected to rise both in the lowlands and at higher altitudes, its life cycle is expected to accelerate, potentially completing in two or three instead of 3 or 4 years. Changes in life cycle length due to high temperature have already been observed in *M. melolontha* populations in Switzerland. During the summer 2022 characterized by a prolonged period of exceptional heat, 3rd instar larvae developed into adults, which started to emerge and fly in September, instead of spring of the following year (Christian Schweizer, Agroscope; personal communication). Reduced life cycle length will increase the frequency of years with serious crop damage and higher losses for farmers and fruit growers. Therefore, it is relevant to regularly monitor the life cycle length of *M. melolontha*, in order to define the most appropriate timing for treatment with *B. brongniartii* biological control products and to assess whether increased temperatures have an effect on the efficacy of these products and on the establishment of *B. brongniartii* in the soil. In addition, because increased temperatures may cause a change in the distribution range of *M. melolontha* and thus causing infestations to reach higher altitudes with less intensively used, high‐biodiversity grasslands, it will be important to monitor its occurrence in newly infested areas and survey potential further damage.

It will be particularly interesting to monitor the development of the distribution range at the border between North and South Tyrol. With rising temperatures, *M. melolontha* might cross the “passo di Resia”, a former landscape barrier. A change in the life cycle length of *M. melolontha* due to global warming, could synchronize previously temporally isolated populations from the south and north of the pass, and directly affect genetic distance between these populations, resulting in increased admixture and gene flow between separate lineages. Because of its sensitivity to temperature fluctuations, which may have direct consequences on the physiology of individuals, their life cycle, and, subsequently, also on the population genomic structure, *M. melolontha* has the potential to become a model insect for monitoring and studying the consequences of climate change in the Alpine region.

In conclusion, the results of this study revealed (1) population genomic structure of *M. melolontha* in the central Alpine region with the existence of two main genetic clusters, i.e., a northwest alpine and a South Tyrol cluster, reflecting evolutionary history and geographic barriers. The “passo di Resia” connecting South and North Tyrol represented a regional contact zone of the two genetic clusters, with a marked genomic differentiation between the northwestern (northwest alpine cluster) and southern (South Tyrol cluster) collections. Although the two collections established from Aosta (northwest Italy) were allocated to the northwest alpine genetic cluster, they showed signs of admixture with the South Tyrolean genetic cluster, indicating shared ancestry. Furthermore, the results showed (2) a significant positive correlation of geographical distance and genetic distance among collections of *M. melolontha*, possibly resulting from limited dispersal capacity. Finally, even though geographic distance was revealed as the main factor influencing genomic structure, the results of the present study also revealed (3) a distinct genetic distance based on temporal isolation (year of flight) of collections, likely as a consequence of reproductive isolation due to temporally synchronized and non‐synchronized swarming flights. This study not only yielded novel data on intraspecific genetic diversity of *M. melolontha* and its connectivity between natural populations, but also important information for current *M. melolontha* monitoring approaches.

## CONFLICT OF INTEREST STATEMENT

The authors declare no conflicts of interest.

## Supporting information


Data S1.

## Data Availability

Raw sequences were submitted to ENA and can be accessed under accession number PRJEB60431.
