# Supplementary material for: Spatial and temporal patterns in the population genomics of the European cockchafer Melolontha melolontha in the Alpine region
Source: Evol Appl. 2023 Sep 1;16(9):1586–97. doi: 10.1111/eva.13588 (PMC10519412; doi:10.1111/eva.13588)
Supplement: Supplementary file 1 — Data S1. [file EVA-16-1586-s001.docx]

Spatial and temporal patterns in the population genomics of the European cockchafer *Melolontha melolontha* in the Alpine region

Supplementary Information

Table of Contents

| **Supplementary Material** | **Page** |
| --- | --- |
| Supplementary Table 1 | 2 |
| Supplementary Table 2 | 3 |
| Supplementary Table 3 | 4 |
| Supplementary Table 4 | 5 |
| Supplementary Figure 1 | 6 |
| Supplementary Figure 2 | 7 |
| Supplementary Figure 3 | 8 |
| Supplementary Figure 4 | 9 |
| Supplementary Figure 5 | 10 |
| Supplementary Figure 6 | 11 |

**Supplementary Table 1.** Sampling sites, year, number of individuals (N) and coordinates of *Melolontha melolontha* collections. A map of sampling site is represented in **Figure 1**. ST: South Tyrol; NT: North Tyrol.

| **Number of the collection** | **Country** | **Site** | **Year of collection** | **N *M. melolontha* ^a^** | **Coordinates latitude WGS84** | **Coordinates longitude WGS84** |
| --- | --- | --- | --- | --- | --- | --- |
| 1 | Switzerland | Eschenz | 2017 | 13 | 47.64186 | 8.85218 |
| 2 | Switzerland | Masein | 2017 | 15 | 46.70192 | 9.42093 |
| 3 | Switzerland | Matten | 2017 | 15 | 46.68555 | 7.86128 |
| 4 | Switzerland | Seewis | 2017 | 14 | 46.99789 | 9.62616 |
| 5 | Switzerland | Strada | 2017 | 13 | 46.86693 | 10.43487 |
| 6 | Switzerland | Tomils | 2017 | 15 | 46.76392 | 9.4404 |
| 7 | Switzerland | Trin Mulin | 2017 | 14 | 46.83169 | 9.34607 |
| 8 | Switzerland | Valzeina | 2017 | 14 | 46.94506 | 9.60554 |
| 9 | Switzerland | Zizers | 2017 | 13 | 46.92481 | 9.57573 |
| 10 | Switzerland | Andhausen | 2018 | 14 | 47.57967 | 9.18453 |
| 11 | Switzerland | Bristen | 2018 | 14 | 46.76556 | 8.70538 |
| 12 | Switzerland | Disentis | 2018 | 15 | 46.69566 | 8.85452 |
| 13 | Switzerland | Falera | 2018 | 13 | 46.79985 | 9.23692 |
| 14 | Switzerland | Ilanz | 2018 | 14 | 46.77473 | 9.21549 |
| 15 | Switzerland | Siat | 2018 | 12 | 46.78566 | 9.16621 |
| 16 | Switzerland | Silenen | 2018 | 13 | 46.78356 | 8.67031 |
| 17 | Switzerland | Valendas | 2018 | 15 | 46.78745 | 9.27761 |
| 18 | Switzerland | Aarenschlucht | 2019 | 13 | 46.71014 | 8.21455 |
| 19 | Switzerland | Bueren | 2019 | 15 | 46.9389 | 8.39378 |
| 20 | Switzerland | Lungern | 2019 | 15 | 46.77879 | 8.15541 |
| 21 | Italy (ST) | Glurns | 2018 | 11 | 46.672067 | 10.559742 |
| 22 | Italy | Aosta-1 | 2019 | 14 | 45.743897 | 7.373035 |
| 23 | Italy | Aosta-2 | 2019 | 15 | 45.714218 | 7.268098 |
| 24 | Italy (ST) | Branzoll | 2019 | 11 | 46.404286 | 11.308656 |
| 25 | Italy (ST) | Kaltern-OG Roen | 2019 | 11 | 46.352351 | 11.262679 |
| 26 | Italy (ST) | Laimburg | 2019 | 15 | 46.381803 | 11.291314 |
| 27 | Italy (ST) | Nals Prissianer Auen | 2019 | 15 | 46.561458 | 11.203442 |
| 28 | Italy (ST) | Passeier-Sandwirt | 2019 | 14 | 46.800521 | 11.244354 |
| 29 | Italy (ST) | Plattl | 2019 | 9 | 46.352211 | 11.305128 |
| 30 | Italy (ST) | Schlanders | 2019 | 10 | 46.627335 | 10.784224 |
| 31 | Italy (ST) | Siebeneich | 2019 | 14 | 46.513761 | 11.268657 |
| 32 | Italy (ST) | Unterrain | 2019 | 14 | 46.497576 | 11.246578 |
| 33 | Austria (NT) | Prutz | 2017 | 15 | 47.077031 | 10.659714 |
| 34 | Austria (NT) | Schoenwies | 2017 | 14 | 47.201453 | 10.670111 |
| 35 | Austria (NT) | Muenster | 2019 | 14 | 47.421711 | 11.840794 |

^a^ Number of individuals per site after mapping and SNP filtering of the ddRADseq data.

**Supplementary Table 2.** Index sequences used for the ddRADseq protocol to construct libraries from *Melolontha* spp. DNA. Forward primer: AATGATACGGCGACCACCGAGATCTACAC-(i5 index)-ACACTCTTTCCCTACACGACG; Reverse primer: CAAGCAGAAGACGGCATACGAGAT-(i7 index)-GTGACTGGAGTTCAGACGTGTGC.

| **Library name** | **i7 index** | **i5 index** |
| --- | --- | --- |
| Reference catalogue | GCTCCGAC | TGACAAGC |
| Library 1 | GGAACGTT | TGACAAGC |
| Library 2 | TGCATTGC | TGACAAGC |
| Library 3 | TCTCATTC | TGACAAGC |
| Library 4 | AAGACGTC | TGACAAGC |
| Library 5 | GGAACGTT | TCCGGATT |
| Library 6 | TGCATTGC | TCCGGATT |
| Library 7 | TCTCATTC | TCCGGATT |
| Library 8 | AAGACGTC | TCCGGATT |
| Library 9 | GGAACGTT | GCTCCGAC |
| Library 10 | TGCATTGC | GCTCCGAC |
| Library 11 | TCTCATTC | GCTCCGAC |
| Library 12 | AAGACGTC | GCTCCGAC |
| Library 13 | GGAACGTT | TGCATTGC |
| Library 14 | TGCATTGC | TGCATTGC |
| Library 15 | TCTCATTC | TGCATTGC |

Supplementary Table 3. Observed (*Ho*) and expected (*He*) heterozygosity per collection of *Melolontha melolontha*. For collection abbreviations see Supplementary Table 1.

| **Number of the collection** | **Observed heterozigosity (*Ho*)** | **Expected heterozigosity (*He*)** |  |
| --- | --- | --- | --- |
| 1 | 0.00013 | 0.00012 |  |
| 2 | 0.00014 | 0.00014 |  |
| 3 | 0.00014 | 0.00014 |  |
| 4 | 0.00013 | 0.00012 |  |
| 5 | 0.00014 | 0.00013 |  |
| 6 | 0.00012 | 0.00012 |  |
| 7 | 0.00012 | 0.00012 |  |
| 8 | 0.00013 | 0.00012 |  |
| 9 | 0.00013 | 0.00013 |  |
| 10 | 0.00012 | 0.00012 |  |
| 11 | 0.00012 | 0.00012 |  |
| 12 | 0.00013 | 0.00013 |  |
| 13 | 0.00012 | 0.00011 |  |
| 14 | 0.00013 | 0.00012 |  |
| 15 | 0.00012 | 0.00012 |  |
| 16 | 0.00013 | 0.00012 |  |
| 17 | 0.00013 | 0.00012 |  |
| 18 | 0.00014 | 0.00013 |  |
| 19 | 0.00014 | 0.00013 |  |
| 20 | 0.00013 | 0.00012 |  |
| 21 | 0.00008 | 0.00007 |  |
| 22 | 0.00016 | 0.00016 |  |
| 23 | 0.00017 | 0.00016 |  |
| 24 | 0.00008 | 0.00008 |  |
| 25 | 0.00008 | 0.00007 |  |
| 26 | 0.00008 | 0.00008 |  |
| 27 | 0.00008 | 0.00007 |  |
| 28 | 0.00008 | 0.00007 |  |
| 29 | 0.00008 | 0.00008 |  |
| 30 | 0.00008 | 0.00007 |  |
| 31 | 0.00008 | 0.00007 |  |
| 32 | 0.00008 | 0.00008 |  |
| 33 | 0.00013 | 0.00013 |  |
| 34 | 0.00012 | 0.00012 |  |
| 35 | 0.00013 | 0.00013 |  |

Supplementary Table 4. DIC and deltaDIC values for the MCMCglmm models employed to investigate the effects of geographic and temporal separation on genetic distances among 35 *Melolontha melolontha* collections.

| **Model** | **DIC** | **DeltaDIC** |
| --- | --- | --- |
| **Null** | -3843.646 | 413.741 |
| **Geo + year** | -4256.387 | 0 |
| **Geo** | -4219.931 | 36.456 |
| **Year** | -3913.733 | 342.654 |

Supplementary Figure 1


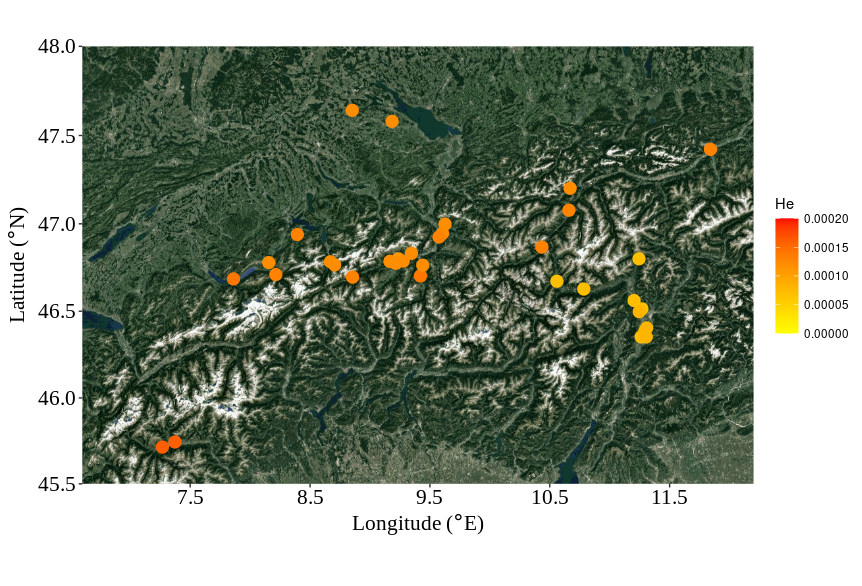


(a)

(b)


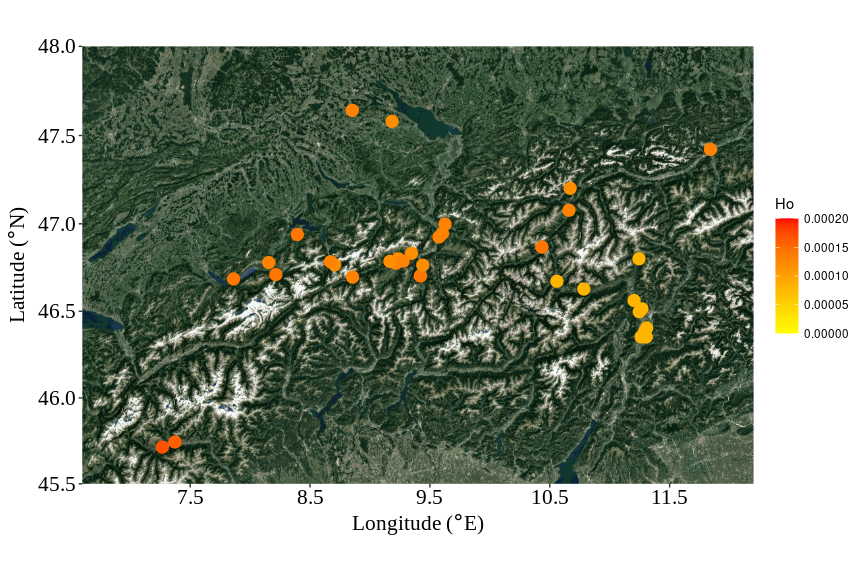


**Supplementary Figure 1.** (**a**) Expected heterozygosity (*He*) and (**b**) observed heterozygosity (*Ho*) of *Melolontha melolontha* collections.

Supplementary Figure 2


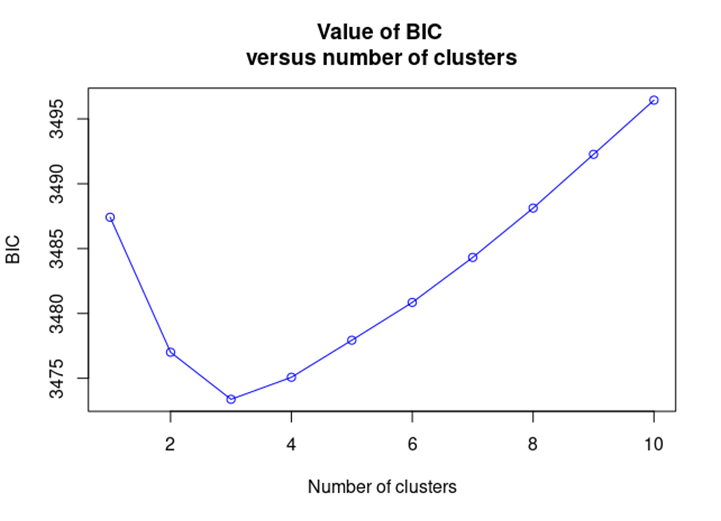


(a)


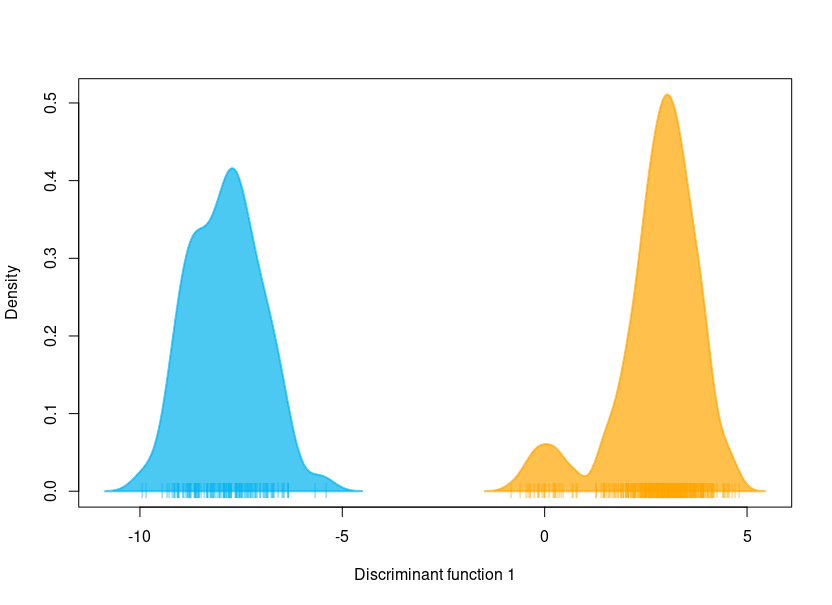

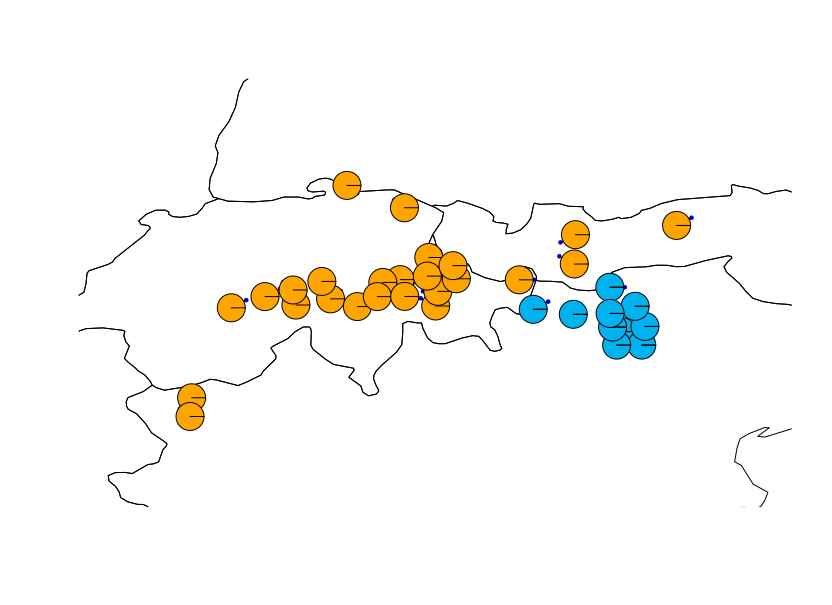


(b)

Axis 1 (97.9)

Axis 2 (2.09%)


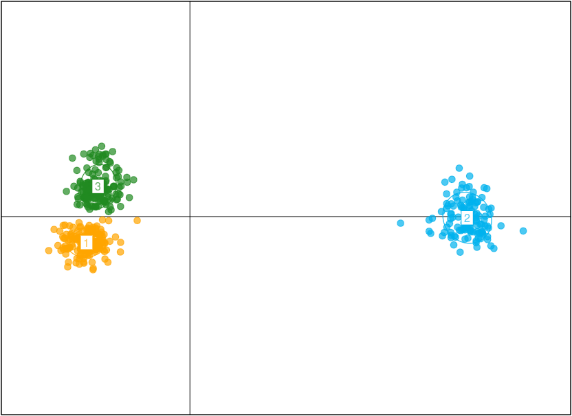

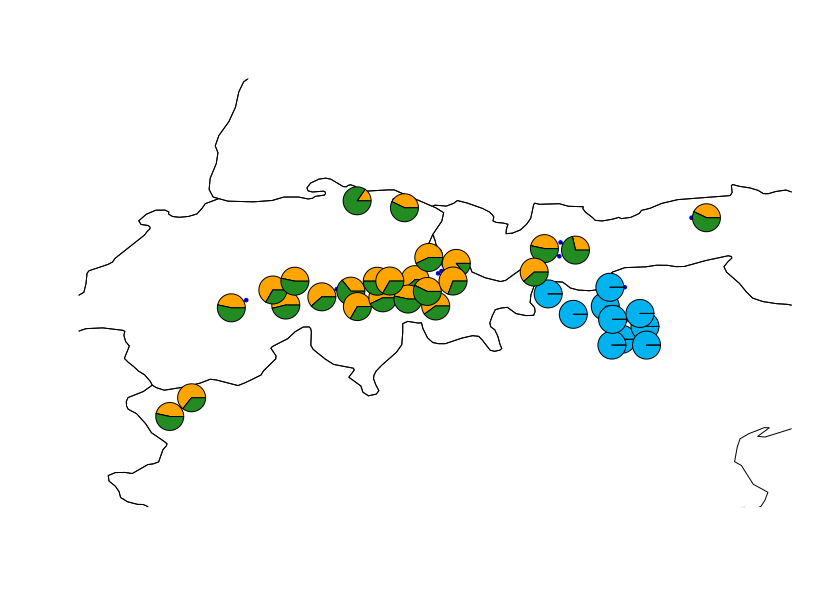


(c)

**Supplementary Figure 2.** Results of a Discriminant Analyses of Principal Components (DAPC) performed including 35 collections of *Melolontha melolontha* (8,358 SNPs, N=475). (**a**) Bayesian Information Criterion (BIC) for K=1-10; DAPC scatterplot calculated across *M. melolontha* individuals and maps of sampling sites with affiliation of individuals for K=2 (**b**) and K=3 (**c**). Colours represent identified genetic clusters.

Supplementary Figure 3


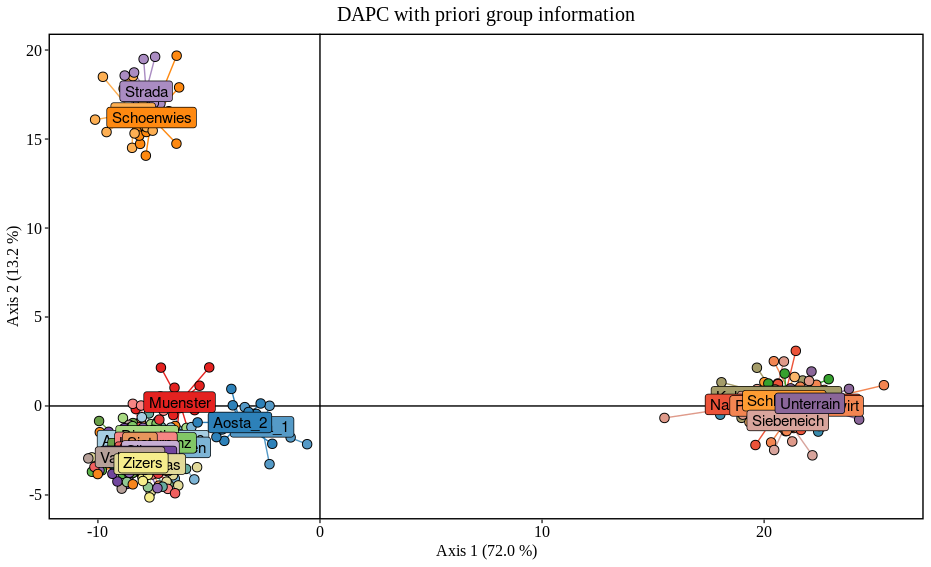


10 South Tyrolean collections

1 eastern Switzerland and 2 North Tyrolean

collections

2 Aosta, 1 North Tyrolean and 19 Swiss

collections

**Supplementary Figure 3.** Results of a Discriminant Analyses of Principal Components (DAPC) analysis performed with a priori group information, including 35 collections of *Melolontha melolontha* (8,358 SNPs, N=475). Colours represent collections.

Supplementary Figure 4


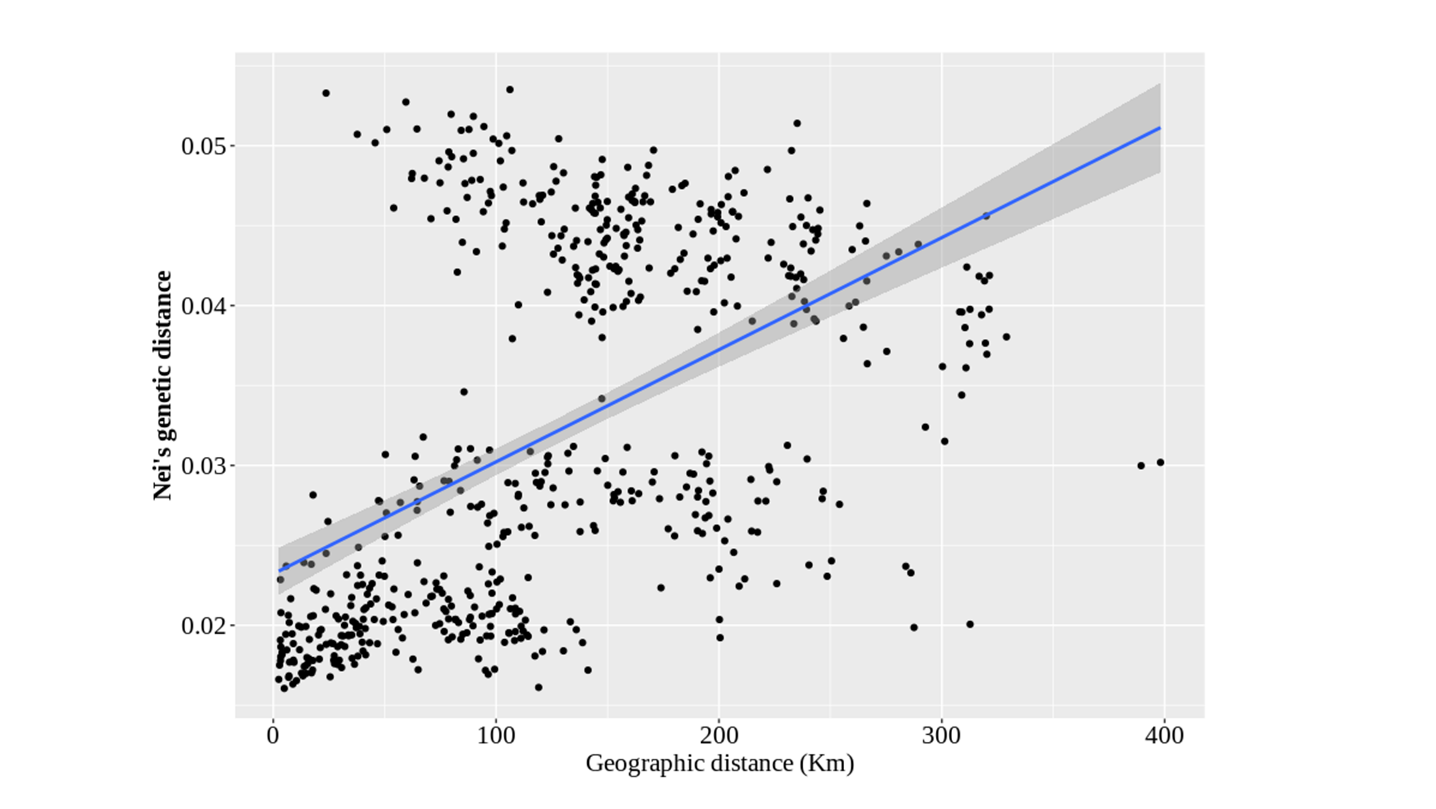


(a)

r: 0.49

p: 0.001


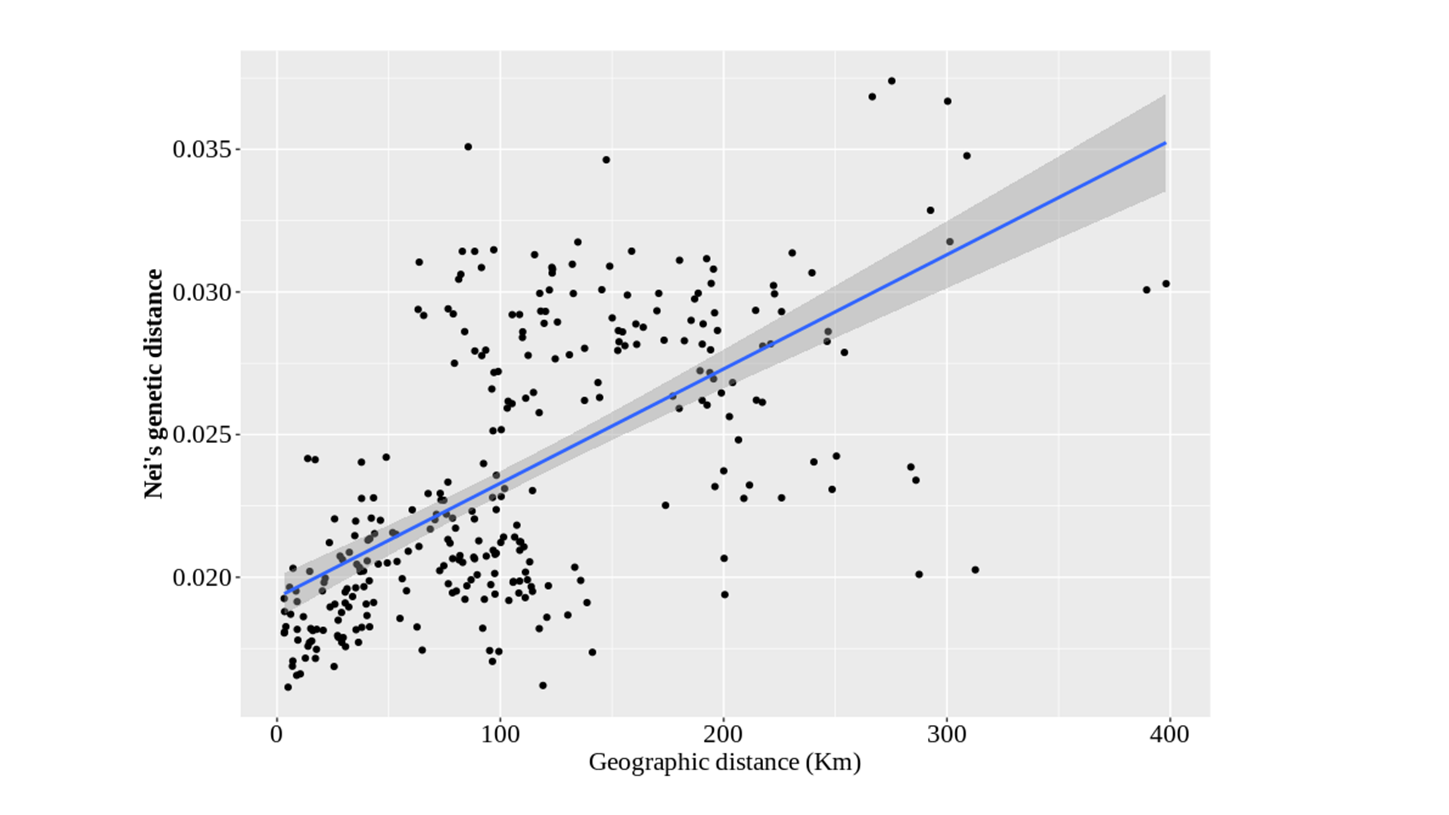


(b)

r: 0.62

p: 0.001

**Supplementary Figure 4.** Isolation by distance (IBD) plots: Nei's genetic distance regressed against geographic distance (Km) in (**a**) the complete *Melolontha melolontha* dataset (8,358 SNPs, N=475) and in (**b**) a *M. melolontha* dataset excluding South Tyrolean collections (8,891 SNPs, N=351).

Supplementary Figure 5


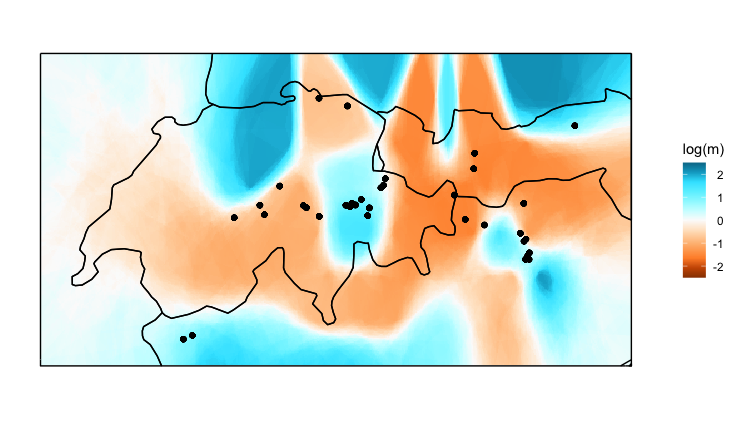


**Supplementary Figure 5.** Results of Estimated Effective Migration Surfaces for posterior mean migration rates of *Melolontha melolontha* over a long evolutionary time scale. The colours illustrate areas of high (blue) and low (orange) migration, respectively. The black dots indicate the 35 sampling sites detailed in **Figure 1**.

Supplementary Figure 6


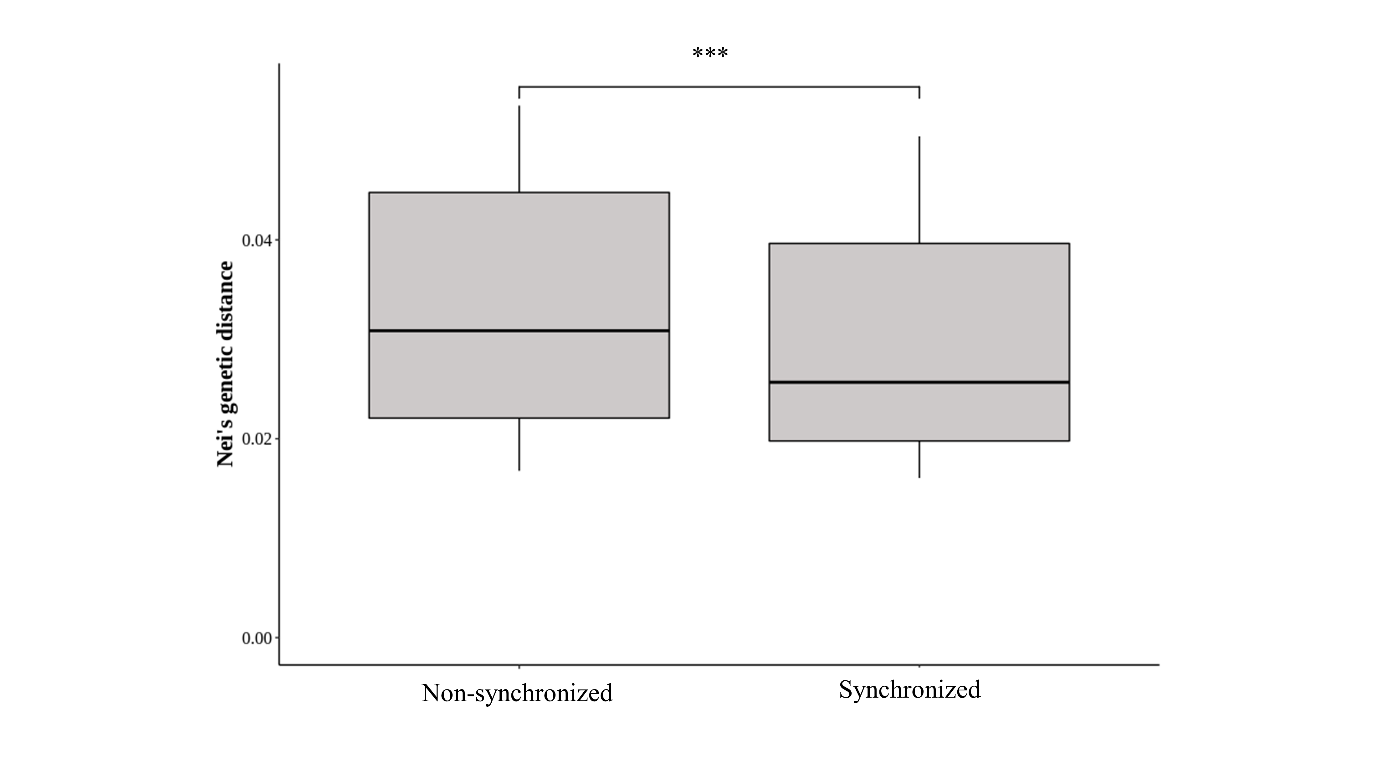


**Supplementary Figure 6.** Box plots of genetic distance between pairs of 35 collections of Melolontha melolontha with a non-synchronized or synchronized swarming flight (t-test; ***: p<0.0001).
